# Decreased Naive and Increased Memory CD4^+^ T Cells Are Associated with Subclinical Atherosclerosis: The Multi-Ethnic Study of Atherosclerosis

**DOI:** 10.1371/journal.pone.0071498

**Published:** 2013-08-23

**Authors:** Nels C. Olson, Margaret F. Doyle, Nancy Swords Jenny, Sally A. Huber, Bruce M. Psaty, Richard A. Kronmal, Russell P. Tracy

**Affiliations:** 1 Department of Pathology, University of Vermont College of Medicine, Burlington, Vermont, United States of America; 2 Departments of Medicine, Epidemiology and Health Sciences, Cardiovascular Health Research Unit, University of Washington, Seattle, Washington, United States of America; 3 Group Health Research Institute, Group Health Cooperative, Seattle, Washington, United States of America; 4 Collaborative Health Studies Coordinating Center, Department of Biostatistics, University of Washington, Seattle, Washington, United States of America; 5 Department of Biochemistry, University of Vermont College of Medicine, Burlington, Vermont, United States of America; University of Tor Vergata, Italy

## Abstract

**Background:**

Adaptive immunity has been implicated in atherosclerosis in animal models and small clinical studies. Whether chronic immune activation is associated with atherosclerosis in otherwise healthy individuals remains underexplored. We hypothesized that activation of adaptive immune responses, as reflected by higher proportions of circulating CD4^+^ memory cells and lower proportions of naive cells, would be associated with subclinical atherosclerosis.

**Methods and Findings:**

We examined cross-sectional relationships of circulating CD4^+^ naive and memory T cells with biomarkers of inflammation, serologies, and subclinical atherosclerosis in 912 participants of the Multi-Ethnic Study of Atherosclerosis (MESA). Circulating CD4^+^ naive cells were higher in women than men and decreased with age (all p-values <0.0001). European-Americans had higher levels of naive cells and lower levels of memory cells compared with African-Americans and Hispanic-Americans (all p-values ≤0.0005). Lower naive/higher memory cells were associated with interleukin-6 levels. In multivariate models, cytomegalovirus (CMV) and *H. Pylori* titers were strongly associated with higher memory and lower naive cells (all p-values <0.05). Higher memory cells were associated with coronary artery calcification (CAC) level in the overall population [β-Coefficient (95% confidence interval (CI))  = 0.20 (0.03, 0.37)]. Memory and naive (inversely) cells were associated with common carotid artery intimal media thickness (CC IMT) in European-Americans [memory: β =  0.02 (0.006, 0.04); naive: β = −0.02 (−0.004, −0.03)].

**Conclusions:**

These results demonstrate that the degree of chronic adaptive immune activation is associated with both CAC and CC IMT in otherwise healthy individuals, consistent with the known role of CD4^+^ T cells, and with innate immunity (inflammation), in atherosclerosis. These data are also consistent with the hypothesis that immunosenescence accelerates chronic diseases by putting a greater burden on the innate immune system, and suggest the importance of prospective studies and research into strategies to modulate adaptive immune activation in chronic disease states such as atherosclerosis.

## Introduction

Clinical, animal model, and epidemiologic research have established roles for activation of innate and adaptive immune responses in atherosclerotic disease [1]. Innate immune responses are characterized by endothelial cell activation in response to perturbations such as accumulation of low density lipoprotein (LDL) particles in the arterial intima. Endothelial activation results in enhanced expression and production of adhesion molecules and chemokines that promote the recruitment of monocytes and T lymphocytes into the arterial wall. Once recruited, monocytes differentiate into macrophages in response to local stimuli, internalize lipoproteins, and form lipoprotein-laden foam cells [1], [2]. In population-based epidemiological studies, increased counts of circulating leukocytes have been associated with subclinical atherosclerosis [3]–[6], and monocyte-derived macrophages and smooth muscle cells are characteristic throughout the atherosclerotic plaque in small-scale clinical studies [7], [8].

Activation of adaptive immune responses have also been implicated in atherogenesis [1]. These responses are initiated upon the recognition of cognate antigen by naive T cells. Antigen recognition results in rapid clonal expansion and differentiation from a naive to an activated, effector subtype, which include helper cells (Th; CD4^+^) and cytotoxic cells (Tc; CD8^+^); CD4^+^ cells include several subtypes including Th1, Th2, Th17, and regulatory T cells (Treg) [9]. Th1 and Th2 subsets are among the best characterized, and are known to promote cell-mediated and humoral responses, respectively [9]. Following resolution of the immune response, a pool of differentiated, antigen-specific memory cells, including Th1 and Th2 subpopulations, persist that are capable of mounting a rapid and enhanced response upon future antigen challenge [9].

Clinical studies have revealed that T cells are abundant in human atherosclerotic lesions. These studies demonstrated that CD4^+^ cells predominate over CD8^+^ cells [7], are mostly memory cell subtypes [10], [11], and display specificity to antigens present in atheromas [12]–[14]. CD4^+^ T cells have been implicated as pro-atherogenic in several experimental mouse models [15], [16], with Th1 responses considered centrally important [1]. Additional CD4^+^ subsets, such as CD4^+^CD28^–^ and Th17, have also recently been implicated as pro-atherogenic [17]–[20].

Despite these observations, our knowledge of the relationships between activation of adaptive immune CD4^+^ T cell responses and atherosclerosis remains limited in human populations, with two relatively small epidemiological studies having reported associations between CD4^+^ effector/memory cells and carotid artery intimal media thickness (IMT) in healthy people [21], [22]. In order to investigate associations of T cell subpopulations with atherosclerosis in an otherwise healthy free-living population, we examined cross-sectional relationships of circulating CD4^+^ naive and memory T lymphocytes with markers of infection, inflammation, and with CC IMT and coronary artery calcification (CAC) in the Multi-Ethnic Study of Atherosclerosis (MESA). We hypothesized that activation of adaptive immune responses, as reflected by greater proportions of circulating memory cells and lesser proportions of naive cells, would be associated with subclinical disease. Additionally, we hypothesized that this same pattern would be associated with age, as well as serologies representing past exposure to pathogens. To date, no multi-ethnic population-based studies have examined these questions.

## Methods

### Ethics Statement

All participants gave written informed consent for participation in the study upon their arrival at the study clinic. The institutional review boards of the six field centers (Johns Hopkins University School of Medicine Institutional Review Boards; Northwestern University Institutional Review Board; University of California, Los Angeles Institutional Review Board; The University of Minnesota Institutional Review Board; Columbia University Medical Center Institutional Review Board; Wake Forest University Health Sciences Institutional Review Board) have approved the study protocol.

### Study Population

MESA is a multi-ethnic, population-based longitudinal epidemiological study initiated in 2000 to investigate the prevalence, correlates and progression of subclinical cardiovascular disease (CVD). A full description of the design and methods for MESA have been reported previously [23]. Briefly, MESA includes 6,814 asymptomatic men and women from four ethnic groups (European-Americans, African-Americans, Hispanic-Americans, and Chinese-Americans) recruited from six field centers in Baltimore MD, Chicago IL, Los Angeles CA, St. Paul MN, New York NY, and Forsyth County NC. Participants were aged 45–84 years and free of clinical CVD during the baseline exam (exam 1) in 2000–2002. Subsequently, exams 2, 3, and 4 occurred in 2002–2004, 2004–2005, and 2005–2007. Results in the present study include data from exam 1 and exam 4, as indicated.

The current study includes a subset of 1000 participants chosen at random for specialized biomarker analysis at the baseline exam. During exam 4 (2005–2007), whole blood samples were collected from this subset and sent overnight to the Laboratory for Clinical Biochemistry Research (LCBR) at the University of Vermont for analysis of cellular phenotypes through the MESA-Inflammation ancillary study. All participants gave informed consent for participation in the study and all procedures were conducted under institutionally approved protocols for human subjects research.

Demographic (age, body mass index (BMI)) and medical (blood pressure (BP), hypertension, diabetes status, smoking status, and medication use) variables are from exam 4. Smoking was defined as never, former (no cigarettes within the past 30 days), or current. Medication use was determined by questionnaire. Type 2 diabetes (T2D) was classified by the 2003 American Diabetes Association criteria, with impaired fasting glucose defined as a fasting glucose between 100–125 mg/dl and diabetes defined as a fasting glucose ≥126 mg/dl [24]. Of those with T2D in the present study (n = 151), 20 were untreated.

### Laboratory Measurements

Lipid and glucose measurements from exam 4 were conducted at the Collaborative Studies Clinical Laboratory at University of Minnesota Medical Center, Fairview (Minneapolis, MN). Lipids were measured on EDTA plasma samples obtained after an overnight fast. Total cholesterol was measured on a Roche Modular P Chemistry Analyzer (Roche Diagnostics, Indianapolis, IN); coefficient of variation (CV) 1.6%. HDL cholesterol was measured using the cholesterol oxidase method (Roche Diagnostics) after precipitation of non-HDL cholesterol with magnesium/dextran; CV 2.9%. Triglycerides were measured using the Roche Modular P Chemistry Analyzer; CV 4.0%. LDL cholesterol was calculated in plasma specimens having a triglyceride value <400 mg/dl using the formula of Friedwald et al. [25]. Serum glucose was measured on the Vitro analyzer (Johnson & Johnson Clinical Diagnostics, Inc, Rochester, NY); CV 1.1%.

Serologies were measured during exam 1 as described previously [26]. Results are reported as ELISA units (EU)/ml (CMV, HSV) or Index Values (*H. pylori*, Hepatitis A); *C. pneumonia* results are reported as 0–4+ microimmunofluorescence units. High sensitivity C-reactive protein (hsCRP) and interleukin-6 (IL-6) were measured during exam 1 as previously described [27]; the analytical CVs were 3.6% and 6.3%, respectively.

### Subclinical Atherosclerosis Measures

Coronary calcification was assessed during exam 1 by chest computed tomography (CT) using either a cardiac-gated electron-beam computed tomography scanner (Chicago, Los Angeles, and New York field centers) or a multidetector computed tomography system (Baltimore, Forsyth County, and St. Paul field centers), as described previously [28]. Each participant was scanned twice over phantoms of known physical calcium concentration. Scans were read by a radiologist or cardiologist at a central center (Los Angeles Biomedical Research Institute at Harbor-UCLA in Torrance, CA). The average Agatston score for the two scans was used for analyses.

Measurements of the internal carotid (IC) and common carotid (CC) arteries were performed during exam 1 by B-mode ultrasonography of the right and left, near and far walls, and images were recorded using a Logiq 700 ultrasound device (General Electric Medical Systems, Waukesha, WI). Maximal IMT of the internal (IC IMT) and common carotid (CC IMT) arteries were measured at a central ultrasound reading center (Department of Radiology, New England Medical Center, Boston, MA) using the mean of the maximum IMT values.

### Analyses of T Cell Phenotypes

During exam 4, blood was drawn into heparinized vacutainers at the MESA field centers and shipped overnight to the LCBR at the University of Vermont in specialized containers that maintained a temperature of 15–30°C. Testing was performed to confirm that the shipping boxes and ambient shipping packs were stable to temperature fluctuations and movement of packages that may occur during overnight shipping. Samples were tested for overnight stability by measuring the cell populations in freshly drawn versus 24-hour post-draw samples subjected to standard shipping procedures in a group of 13–20 local apparently healthy volunteers (Figure S1). Regression slopes comparing fresh versus 24-hour post-draw cell measurements were 1.06 for naive cells, and 1.05 for memory cells, and their respective Pearson correlation coefficients were 0.97 and 0.97, indicating that results were similar between the two samples.

Collection tubes were mixed gently on a shaker for 10 minutes prior to the start of the protocol. An aliquot of whole blood (100 μl) was incubated with allophycocyanin-cyanine dye Cy7 (APC-Cy7)-conjugated anti-CD4 (BD Biosciences, San Jose, CA), fluorescein isothiocyanate (FITC)-conjugated anti-CD45RO (BD Biosciences), and R-phycoerythrin cyanine dye Cy5.5 (PE-Cy5.5)-conjugated anti-CD45RA (Invitrogen, Carlsbad, CA) for 20 minutes in the dark, according to the manufacturers' instructions. APC-Cy7-conjugated mouse IgG1 κ (BD Biosciences), FITC-conjugated mouse IgG1 κ (BD Biosciences), and PE-Cy5.5-conjugated mouse IgG2b (Invitrogen) were analyzed as isotype matched controls.

Red blood cells were lysed by adding Pharm Lyse (BD Biosciences) to each tube, vortexing for 1 minute and incubating in the dark for 15 minutes. Cells were centrifuged at 453 x g for 15 minutes. Supernatants were decanted and the cell pellets were resuspended in 1 mL of Pharm Lyse, vortexed, incubated for 5 minutes, and centrifuged as above to eliminate residual red blood cells. The cell pellets were resuspended in 0.4 mL of 1% paraformaldehyde and maintained at 4°C protected from light until evaluated.

**Figure 1:**
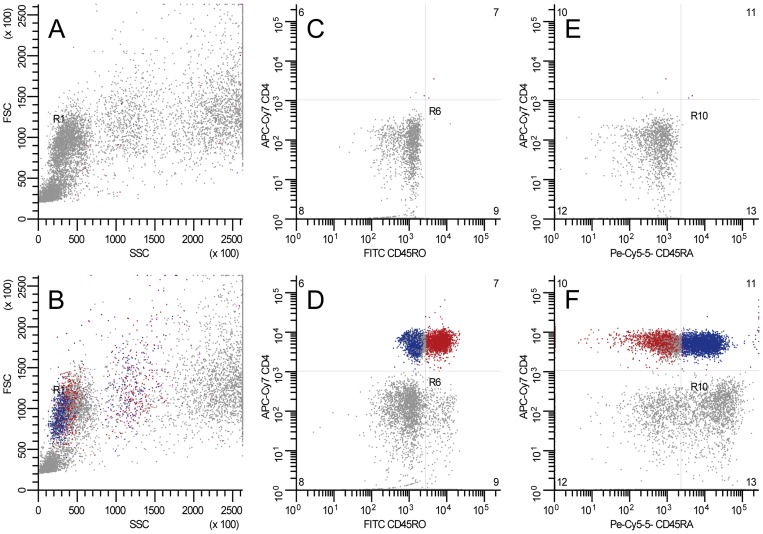
Typical flow cytometric data for lymphocyte, CD4^+^ memory, and CD4^+^ naive cell populations. At least 30,000 lymphocytes were evaluated and were gated based on their forward (FCS; X-axis) and side (SCS; Y-axis) scatter (Panels A&B). Memory and naive cell subpopulations were gated by positive surface staining for CD4 (Y-axis, panels C–F); memory cells were gated by positive surface staining for CD45RO (X-axis, panels C&D); naive cells were gated by positive surface staining for CD45RA (X-axis, panels E&F). Typical data from respective isotype controls (Panels A, C, and E) and fluorescently labeled samples (Panels B, D, and F) are shown.

Cells were analyzed by flow cytometry using an LSR II flow cytometer (BD Biosciences) with a single excitation wavelength (488 nm) and band filters for APC-Cy7 (780/60), FITC (530/30 nm), and PE (575/26 nm). At least 30,000 lymphocytes were evaluated, and were gated based on their forward and side scatter. Positive fluorescent controls were used to set machine compensation, and background staining was determined using the negative isotype controls. T helper lymphocytes were gated by positive surface staining for CD4 and were expressed as a percentage of gated lymphocytes. Memory and naive subpopulations were gated by positive surface staining for CD45RO and CD45RA, respectively. A prior study in our laboratory demonstrated concordance between percentages of naive and memory cells gated as CD4^+^CD45RA^+^ (or CD4^+^CD45RO^+^) and re-gated as CD4^+^CD45RA^+^RO^−^ (or CD4^+^CD45RO^+^CD45RA^−^); the *R* ^2^ values were 0.92 and 0.90 for % naive and % memory, respectively (unpublished data). Given the strong correlation between these two methods, naive and memory cells were expressed as the percentage of CD4^+^ cells that were CD4^+^CD45RA^+^ (naive) and CD4^+^CD45RO^+^ (memory) in the present study. A typical set of flow cytometric data for lymphocyte, CD4^+^ naive cell, and CD4^+^ memory cell populations is illustrated in Figure 1.

Comprehensive assay validation was performed as previously described [29] by enlisting 14 local volunteers who donated samples prior to the commencement of the study and periodically throughout the study, which allowed us to assess biovariability. Three analytic CVs for the protocol were calculated using a nested analysis of variance as described previously [30]; analytical (CVa), within-subject (CVi), and between-subject (CVg). In cases where analysis was done only in singleton, no CVa was calculated. The Index of Individuality (II), which is the ratio of the within-individual variation (CVi) to the between-subject variation (CVg), was calculated. These biovariability data are summarized in Table S1. Assays with II values <1 are generally considered acceptable in population studies [30]. The IIs for %memory and %naive were 0.45 and 0.44, respectively. These values are very similar to cholesterol (II = 0.44), a widely accepted biomarker [30].

### Statistical Analyses

All statistical analyses were conducted using the Statistical Analysis System (SAS, version 9.2; SAS Institute, Inc., Cary, NC). Naive and memory cells were analyzed as proportions of CD4^+^ cells. We have chosen to express the data as percentages rather than cell counts to help account for potential transient changes in leukocyte numbers. Analysis of variance or Pearson correlations were used to test for associations of T cell subpopulations and demographic variables, and with markers of inflammation and infection. For serological analyses, titers or index values were used.

Standardized backward elimination regression was used to develop multivariate models of naive and memory proportions. These analyses included age, gender, race/ethnicity, seasonality, BMI, IL-6, and CMV and *H. pylori* titers as the independent starting variables. Only significant variables (p≤0.05) were retained in the final models.

Minimally adjusted and backward elimination regression methods were used to evaluate associations of T cell subpopulations and subclinical disease measures. Presence of CAC was evaluated by comparing those with CAC = 0 versus those with CAC >0 using relative risk regression. CAC level was assessed using natural logarithm (ln)-transformed Agatston scores as the dependent variable in those participants with a score >0. Minimally adjusted models included age, gender, race/ethnicity and naive or memory proportions as the independent variables. Additional analyses included demographics (age, gender, race/ethnicity), markers of inflammation and infection (IL-6, CMV and *H. pylori* titers), traditional CVD-related variables (BMI, systolic BP, use of BP lowering medication, smoking status, total-cholesterol, HDL-cholesterol, use of lipid lowering medication, and diabetes status), and memory or naive proportions, as the candidate starting variables. CC IMT and IC IMT were analyzed in the entire study population and stratified by race/ethnicity. Sensitivity of the findings to outliers was assessed by rerunning the analyses using robust regression, for which results were essentially the same.

## Results

Characteristics of the study population are shown in Table 1. CD4^+^ naive and memory cell proportions were normally distributed (Figure S2). The mean (SD) values in the overall population were 28.3% (14.2%) and 53.9% (15.2%), and ranged from 2.6%–78.0% and 15.9%–92.6% for naive and memory cells, respectively. Mean (SD) proportions of circulating naive cells were higher in women (30.1% (14.2%)) than men (26.4% (14.0%)) (p<0.0001, adjusted for age and race/ethnicity). Memory cell values were similar between genders [53.7% (15.3%) and 55.6% (15.0%) for women and men, respectively].

**Table 1:** Characteristics of the MESA sample population being studied.

Variable	MESA-Inflammation
*n*	912
Age, years (mean, SD)	65.7 (9.9)
Gender (Men, *n* (%))	437 (48)
Race/Ethnicity (*n*, %)	
European-American	396 (43)
African-American	187 (21)
Hispanic-American	234 (26)
Chinese-American	95 (10)
BMI, kg/m^2^ (mean, SD)	28.6 (5.6)
Hypertension (*n*, %)	448 (49)
Smoking Status (*n*, %)	
Never	399 (44)
Former	425 (47)
Current	83 (9)
Lipid Status (mean, SD)	
Total-Cholesterol, mg/dl	187.6 (37.3)
LDL-Cholesterol, mg/dl	110.6 (33.5)
HDL-Cholesterol, mg/dl	52.6 (15.0)
Triglycerides, mg/dl	123.1 (72.2)
Inflammatory Biomarker^*^	
CRP, mg/l (median, 25^th^, 75^th^)	1.97 (0.86, 4.43)
IL-6, pg/ml (median, 25^th^, 75^th^)	1.15 (0.75, 1.79)
Lymphocytes (mean, SD)	
CD4^+^ (% of lymphocytes in whole blood)	39.9 (10.3)
Naive (% of CD4^+^ cells)	28.3 (14.2)
Memory (% of CD4^+^ cells)	53.9 (15.2)
Serology^*^ (% seropositive)	
CMV	75
* H. pylori*	44
Hepatitis A	55
HSV	84
* C. pneumoniae*	74
Type 2 Diabetes (*n*, %)	151 (17)
Atherosclerosis Status^*^	
Presence of Coronary Calcification (*n*, %)	434 (48)
CAC in Positives, AU (median, 25^th^,75^th^)	72.3 (20.8, 299.4)
IMT, common carotid, mm (mean, SD)	0.87 (0.19)
IMT, internal carotid, mm (mean, SD)	1.03 (0.54)
Clinical CVD at Exam 4 (*n*)**	32

Data are from MESA exam 4 (2005–2007) unless otherwise noted. *: Data are from MESA baseline (exam 1; 2000–2002). **: Includes all participants with incident cardiovascular events (myocardial infarction, resuscitated cardiac arrest, definite or probable angina, and stroke) from baseline through the start of exam 4. AU: Agatston units; BMI: Body mass index; CAC: Coronary artery calcification; CMV: Cytomegalovirus; CRP: C-reactive protein; CVD: Cardiovascular disease; HSV: Herpes simplex virus; IL-6: Interleukin-6; IMT: Intimal media thickness.

Proportions of circulating naive cells decreased with increased age (p<0.0001 for trend). Mean (SD) proportions of circulating naive cells were 31.4% (14.1%) in the lowest age quartile (45–54 years; *n* = 140) compared with 24.8% (14.3%) in the highest quartile (75–84 years; *n* = 197; p<0.0001 adjusted for gender and race/ethnicity). Results were similar in analyses that additionally adjusted for CMV seropositivity. No statistically significant associations were observed between memory cell proportions and age.

European-Americans had higher levels of naive cells compared with African-Americans and Hispanic-Americans [30.9% (13.8%), 25.8% (13.8%), and 24.8% (13.8%), respectively; both p-values ≤0.0005 compared to European-Americans, adjusted for age and gender]. European-Americans had lower levels of memory cells compared with African-Americans and Hispanic-Americans [50.6% (14.9%), 55.8% (14.9%), and 58.1% (14.9%), respectively; both p-values ≤0.0005 compared to European-Americans, adjusted as above]. Chinese-Americans were similar to European-Americans [naive = 30.1% (13.8%); memory = 54.2% (14.9%)]. Additional adjusting for CMV seropositivity partially attenuated the results. The adjusted means (p-value compared to European-Americans) for naive cells were 26.4%, 22.1% (p = 0.02), and 22.3% (p = 0.02) for European-Americans, African-Americans, and Hispanic-Americans, respectively. For memory cells these values were 49.7%, 53.5% (p = 0.06), and 55.0% (p = 0.002).

Naive and memory proportions were correlated with the inflammatory biomarkers IL-6 and CRP, and with BMI (Table 2). In analyses stratified by race/ethnicity, adjusting for gender, stronger associations were seen for IL-6 and naive cells (*r* = −0.20; p<0.0001) and memory cells (*r* = 0.26; p<0.0001) in European-Americans only. No significant correlations were observed for either subpopulation and with variables representing common CVD risk factors, including blood pressure measures, smoking status, presence of T2D, and lipid measures (total-cholesterol, LDL-cholesterol, HDL-cholesterol, triglycerides) (Table 2).

**Table 2:** Pearson correlation coefficients for CD4^+^ naive and memory cell subpopulations with biomarkers of inflammation, CVD risk, and infection.

Variable	%Naive	P value	%Memory	P value
*Inflammation*				
CRP (mg/l)	−0.07	0.03	0.06	0.05
IL-6 (pg/ml)	−0.12	0.0004	0.15	<0.0001
*CVD*				
BMI (kg/m^2^)	−0.12	0.0003	0.08	0.02
Systolic blood pressure (mmHg)	−0.03	ns	0.02	ns
Diastolic blood pressure (mmHg)	−0.01	ns	−0.007	ns
Total-cholesterol (mg/dl)	0.05	ns	−0.03	ns
LDL-cholesterol (mg/dl)	0.04	ns	−0.03	ns
HDL-cholesterol (mg/dl)	0.03	ns	−0.04	ns
Triglycerides (mg/dl)	−0.01	ns	0.04	ns
*Serology*				
CMV	−0.17	<0.0001	0.17	<0.0001
* H. pylor*	−0.12	0.001	0.11	0.003
Hepatitis A	0.08	0.04	−0.03	ns
HSV	−0.07	ns	0.07	ns

Antibody titers or index values were used for serologic analyses. Analyses are adjusted for age, gender, and race/ethnicity. BMI: Body mass index; CMV: Cytomegalovirus; CRP: C-reactive protein; CVD: Cardiovascular disease; HSV: Herpes simplex virus; IL-6: Interleukin-6; ns: non-significant; p>0.05.

Adjusting for age, gender, and race/ethnicity, naive proportions were negatively correlated with past exposure to CMV and *H. Pylori*, with weak positive correlations observed with Hepatitis A. Memory cell values were positively correlated with CMV and *H. Pylori.* No associations were observed between either subpopulation and HSV or *C. pneumonia* (Table 2).

To identify potential predictors of CD4^+^ naive and memory cell proportions, standardized backwards elimination multivariate models were developed (Table 3). For naive cells, the main independent variables were, gender, season, CMV and *H. Pylori* titers, with lesser contributions from age, IL-6 and BMI (*R* ^2^ for the final model = 0.15). For memory cells, the main independent variables were Hispanic-American race/ethnicity, and CMV and *H. Pylori* titers, with lesser contributions from gender and IL-6 (*R* ^2^ for the final model = 0.12).

**Table 3:** Regression models for CD4^+^ naive and memory cell subpopulations.

Variable (increment)	%Naive		%Memory	
	β Coef.	P value	β Coef.	P value
Age (10 yr. increase)	−1.25	<0.05	−0.47	ns
Gender (male vs. female)	−4.2	<0.0001	2.81	<0.05
Race/Ethnicity(vs. European-American)				
Chinese-American	1.91	ns	1.11	ns
African-American	−1.97	ns	2.61	ns
Hispanic-American	−1.69	ns	4.33	<0.005
Season (vs. Winter)				
Spring	2.11	ns	0.01	ns
Summer	4.07	<0.05	3.26	ns
Fall	7.21	<0.0001	0.76	ns
BMI (5.6 kg/m^2^)	−1.26	<0.05	0.54	ns
IL-6 (1.2 pg/ml)	−1.49	<0.05	2.26	<0.0001
CMV (vs. 0.0 EU/ml)				
0.1 – 99.9 EU/ml	−2.91	<0.05	4.71	<0.005
100 – 199.9 EU/ml	−6.15	<0.0001	8.11	<0.0001
200 – 299.9 EU/ml	−7.05	<0.005	7.29	<0.005
*H. pylori* (vs. <0.9)				
0.9–6.9	−3.44	<0.005	2.68	<0.05
6.9–13.9	−6.60	<0.05	3.99	<0.05
Model R^2^	0.15		0.12	

Backward elimination regression was used to develop multivariate models for CD4^+^ T cell subpopulations. Independent variables were divided by their standard deviations (shown in parentheses). Age, gender, race/ethnicity, seasonality, BMI, IL-6, and CMV and *H. pylori* titers were included as the candidate starting variables. Only significant variables (p<0.05) were retained in the final models. BMI: Body mass index; CMV: Cytomegalovirus; IL-6: Interleukin-6; ns: non-significant.

In minimally adjusted (age, gender, and race/ethnicity) regression models, there was no association of either T cell subpopulation with the presence of CAC (CAC = 0 versus CAC >0; data not shown). Memory cell proportions were significantly associated with CAC level (i.e., in those with CAC >0), in minimally adjusted regression models (β = 0.19±0.09; p = 0.03). Memory cell proportions remained significantly associated with CAC level in backward elimination models that included demographics, traditional CVD-related variables (BMI, systolic BP, use of BP lowering medication, smoking status, total-cholesterol, HDL-cholesterol, use of lipid lowering medication, and T2D status), and biomarkers of inflammation and infection (IL-6, CMV and *H. pylori* titers) as starting candidates (Table 4). Naive cell proportions were inversely associated with CAC level, but these associations were not statistically significant [minimally adjusted model: β = −0.16±0.09; p = 0.08; fully adjusted model: β = −0.16±0.09; p = 0.09].

**Table 4:** Final regression model for coronary artery calcification.

Independent Variable (increment)	lnCAC (*n* = 434)
	β-Coefficient (95% CI)
Age (10 yr. increase)	0.82 (0.65, 1.0)
Gender (male vs. female)	0.70 (0.35, 1.0)
Lipid-lowering medication	0.65 (0.31, 1.0)
Diabetes Status (vs. normal)	
Impaired fasting glucose	0.46 (0.06, 0.86)
Diabetes (untreated)	ns
Diabetes (treated)	ns
CD4^+^ Memory cells (15.2%)	0.20 (0.03, 0.37)
Model R^2^	0.21

Backward elimination regression was used to develop multivariate models for coronary artery calcification (CAC) level. CAC was analyzed using the ln-agatston score in individuals with a score >0. Independent variables were divided by their standard deviations (shown in parentheses). The candidate starting variables were: age, gender, race/ethnicity, IL-6, BMI, systolic BP, use of BP lowering medication, smoking status, total-cholesterol, HDL-cholesterol, use of lipid lowering medication, type 2 diabetes status, CMV and *H. pylori* titers, and CD4^+^ memory cell proportions or, in separate analyses, CD4^+^ naive cell proportions. Only significant variables (p<0.05) were retained in the final model to obtain the model's R^2^. ns: non-significant.

In minimally adjusted analyses, no significant associations were observed with memory or naive cell proportions and CC IMT in the overall study population. In analyses stratified by race/ethnicity (adjusted for age and gender), both T cell populations were significantly associated with CC IMT in European-Americans [β = 0.02±0.008 (p = 0.04); β = −0.02±0.007 (p = 0.01) for memory cells and naive cells, respectively]. These associations remained after including variables related to inflammation/infection and CVD (Table 5), as described above for CAC. No significant associations were observed in the other racial/ethnic groups. No statistically significant associations of either T cell subpopulation were observed with IC IMT.

**Table 5:** Final regression models for common carotid intimal media thickness in European-Americans.

Independent Variable (increment)	CC IMT (*n* = 396)
	β-Coefficient (95% CI)
Age (10 yr. increase)	0.11 (0.09, 0.13)
BMI (5.6 kg/m^2^)	0.03 (0.01, 0.04)
CD4^+^ Memory cells (15.2%)	0.02 (0.006, 0.04)
CD4^+^ Naive cells (14.2%)	−0.02 (−0.004, −0.03,)
Model R^2^	0.36

Multivariate regression was performed as described in Methods with common carotid intimal media thickness (CC IMT) as the dependent variable. CD4^+^ naive and memory cells were analyzed in separate models. Analyses were stratified by race/ethnicity; results from European-Americans are presented.

## Discussion

In this multi-ethnic, cross-sectional epidemiological study, we have demonstrated that a) chronic adaptive immune activation, as reflected by higher circulating CD4^+^ memory cells and lower circulating CD4^+^ naive cells, is associated with the degree of subclinical atherosclerosis; b) CMV and *H. pylori* serological titers were identified as the main predictors of memory and naive subpopulations, suggesting a potential role of these pathogens in chronic immune activation and inflammation, and through these pathways, chronic diseases such as atherosclerosis.

The positive associations found in our study between CD4^+^ memory cells, which reflect previous activation of adaptive immune responses, and measures of subclinical atherosclerosis are consistent with clinical research [10], [11], mouse model data [15], [16], and a limited number of population-based epidemiology studies [21], [22]. In these latter studies, increased counts of CD4^+^ memory cells were positively associated with the mean CC IMT in 557 apparently healthy elderly Japanese men [21]. Circulating proportions of CD4^+^ effector memory cells were also recently associated with CC IMT in 183 otherwise healthy Italian participants [22].

The current study extends findings to include both CC IMT and CAC, and memory and naive cells in a multi-ethnic population. Coronary artery calcification is a characteristic feature of atherosclerosis and is predictive of future cardiovascular events [31]. Although the mechanism of arterial calcification is incompletely understood, several inflammatory mediators secreted by T cells, such as interferon-γ (IFN-γ) and tumor necrosis factor-α (TNF-α), are thought to play important roles [32]. Interestingly, activated T cells have been demonstrated to influence bone remodeling through their expression of the TNF receptor family molecule, receptor activator of NF-kB ligand (RANK-L), a key regulator of osteoclastogenesis [33]. Circulating T cell populations have also recently been associated with cases of calcific aortic stenosis [34]. Collectively, these data suggest that certain T cell subsets may play an underexplored role in calcification and/or bone remodeling. This is the first report to describe associations between CD4^+^ memory cells and CAC, and will require replication in other study populations.

The associations of memory T cell populations with subclinical atherosclerosis in this and other studies [21], [22], suggests T cell activation may modulate disease progression, and several potential pathophysiological mechanisms have been identified using mouse models. Among the best characterized are Th1 cell responses, which are facilitated predominately through their secretion of IFN-γ [1]. In mouse models of atherosclerosis, IFN-γ has been demonstrated to recruit T cells and macrophages to atherosclerotic lesions, increase macrophage uptake of lipids, and contribute to plaque destabilization [1]. As polarized Th1 cells rather than naive (Th0) cells are the primary T cell population producing IFN-γ, are known to comprise a portion of the T cell memory pool [35], and have recently been associated with subclinical atherosclerosis in MESA [29], it is hypothesized that the associations of memory cells with subclinical atherosclerosis in the present study reflect activation and polarization of Th1-mediated responses. In addition, Th17 cells have also been implicated as pro-atherogenic, and could represent a portion of the memory pool [17], [18]. It is, however, important to note that our cross-sectional study design cannot establish causality, and prospective studies are needed.

Counter to pro-inflammatory effector/memory T cells, Tregs function as negative immune regulators serving to maintain immunological tolerance and suppress effector T cell responses. There is significant evidence that Treg cells are protective in mouse models of atherosclerosis [36], and while certain lines of evidence have implicated a protective role in human CVD [37], [38], associations have been inconsistent [39]. Collectively, these data may suggest an imbalance between effector/memory T cells and Tregs in the progression of atherosclerotic disease [36]. Differences in effector/memory: Treg ratios may be one explanation for the lack of association between effector/memory cells with subclinical atherosclerosis in previous studies [40], [41].

As opposed to direct contributions of CD4^+^ T cells towards atherosclerosis, associations of chronic CD4^+^ T cell activation with atherosclerosis may reflect persistent immune activation and inflammation resultant from chronic pathogenic burden. The consequences of latent or chronic pathogen infection, characterized by reduced naive T cells and increased memory T cells, include: accumulation of antigen-specific T cells leading to restricted T cell receptor diversity and a limited capacity to mount responses against previously unencountered antigens [42]; altered regulation of innate immune responses by T helper and Treg cells; and loss of thymic output leading to immunosenescence [43]. Collectively, these outcomes would serve to promote innate-mediated inflammation, and may be an indirect mechanism whereby chronic adaptive immune activation potentiates atherosclerosis; our finding of an association between higher memory/lower naive and IL-6 levels, especially in European-Americans, supports this hypothesis.

This is the first study to report that CMV is a major predictor of CD4^+^ naive and memory cell proportions in a large multi-ethnic population, and is consistent with a role of CMV in chronic adaptive immune activation, restriction of the T cell repertoire [42], [44], and as a driving force in human immunosenescence [43]. The findings that CMV-specific T cell responses are independently associated with subclinical atherosclerosis in HIV infected patients [45] supports the hypothesis that CMV may play a role in the development of atherosclerosis by promoting chronic immune activation and inflammation, and may have implications in chronic disease states beyond atherosclerosis.

Our results additionally implicate *H. pylori* as an important and, as yet, underappreciated, determinant of CD4^+^ naive and memory subpopulations in multi-ethnic human populations. *H. pylori* infected individuals have been demonstrated to have impaired CD4^+^ memory responses [46], potentially due to the suppression of effector/memory function by CD4^+^CD25^+^ Treg cells [46], [47]. *H. pylori* has also been reported to impair Th1 development by modulating dendritic cell function [48], [49]. This suggests that *H. pylori* infection affects atherogenesis by impairing adaptive immunity, and increasing innate immune activity, rather than by modulation of CD4^+^ T helper cell polarization. Additional investigations will be required in order to confirm the present findings.

In addition to the associations of naive and memory cells with infections pathogens, we observed decreased naive cell proportions with increased age. These collective findings are consistent with reports of declining immune function associated with the advancement of age and with CMV infection (i.e., immunosenescence) [44], [50]. The use of additional markers such as CD28^−^ and CD57^+^ that are characteristic of immunosenescent cell populations, and/or measurements of thymic output such as T cell recombination excision circles (sjTREC) [51], are warranted in future population-based studies.

Strengths of the present study include the diversity and health status of the study population, which includes four ethnicities across a wide age range, from geographically distinct regions in the U.S.; validated flow cytometric measures done with a comprehensive quality assurance/quality control program; and the rich MESA data base of demographic, molecular, and imaging data. Limitations include the cross-sectional study design which restricts our ability to determine the temporal relationships between subclinical atherosclerotic and cellular measures; and that some of the outcome variables, such as the measures of subclinical CVD, were not from exam 4, biasing our analyses to the null.

In summary, we have measured circulating CD4^+^ naive and memory cells in a multi-ethnic population and demonstrated that the degree of chronic adaptive immune activation, as estimated by lower circulating naive cells and higher memory cells, is associated with the extent of subclinical atherosclerosis as estimated by both carotid ultrasonography and coronary MRI imaging. These results are consistent with the known role of CD4^+^ T cells, especially Th1 cells, in atherosclerosis and with the hypothesis that immunosenescence accelerates chronic diseases by putting a greater burden on the innate immune system. These data suggest the importance of prospective studies and research into strategies to modulate adaptive immune activation in chronic disease states such as atherosclerosis.

## Supporting Information

Figure S1 **Linear Regression Analyses of Cellular Measurements in Fresh Whole Blood versus 24-Hour Post-Draw Samples.** The X-axis represents values from freshly processed whole blood and the Y-axis represents values from 24-hour post-draw processing of whole blood. Panel A, %CD4^+^ naive cells; Panel B, %CD4^+^ memory cells.
(TIF)

Figure S2 **Distributions of CD4^+^ Naive and Memory Cells in MESA-Inflammation.** Distributions of CD4^+^ naive (A) and memory (B) cells are shown in the overall study population. Y-axis: count; X-axis: value.
(TIF)

Table S1 **Biovariability Measures for Cellular Phenotypes.**
(DOCX)
