# Supplementary figures and images for: Decreased Naive and Increased Memory CD4+ T Cells Are Associated with Subclinical Atherosclerosis: The Multi-Ethnic Study of Atherosclerosis
Source: PLoS One. 2013 Aug 23;8(8):e71498. doi: 10.1371/journal.pone.0071498 (PMC3751895; doi:10.1371/journal.pone.0071498)

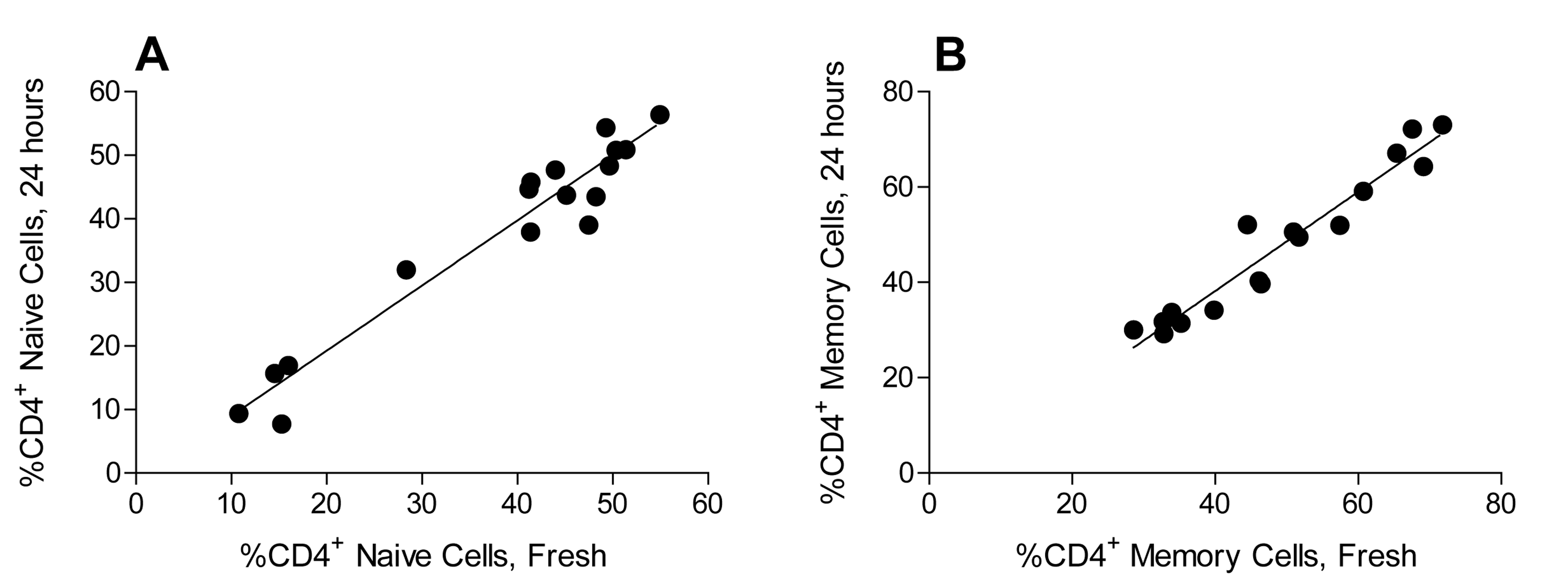

Supplement: Figure S1 — Linear Regression Analyses of Cellular Measurements in Fresh Whole Blood versus 24-Hour Post-Draw Samples. The X-axis represents values from freshly processed whole blood and the Y-axis represents values from 24-hour post-draw processing of whole blood. Panel A, %CD4+ naive cells; Panel B, %CD4+ memory cells. (TIF) [file pone.0071498.s001.tif]

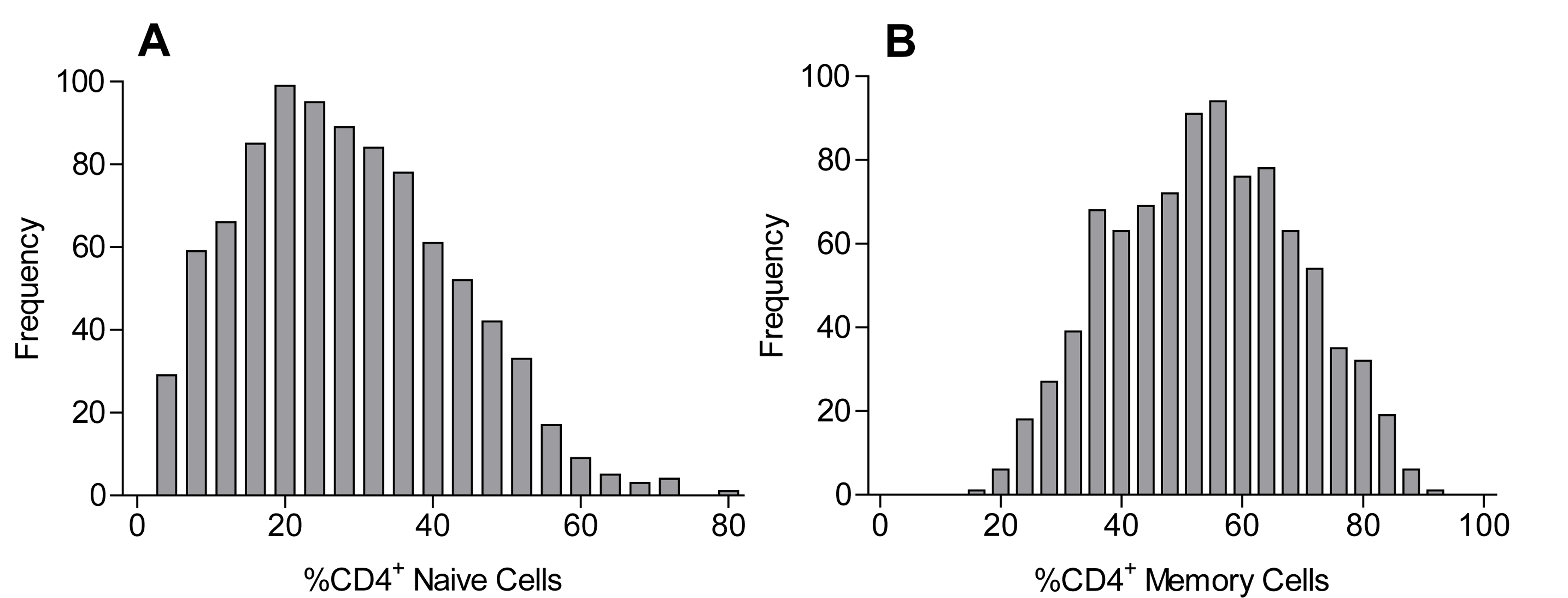

Supplement: Figure S2 — Distributions of CD4+ Naive and Memory Cells in MESA-Inflammation. Distributions of CD4+ naive (A) and memory (B) cells are shown in the overall study population. Y-axis: count; X-axis: value. (TIF) [file pone.0071498.s002.tif]
