# Supplementary material for: Decreased Naive and Increased Memory CD4+ T Cells Are Associated with Subclinical Atherosclerosis: The Multi-Ethnic Study of Atherosclerosis
Source: PLoS One. 2013 Aug 23;8(8):e71498. doi: 10.1371/journal.pone.0071498 (PMC3751895; doi:10.1371/journal.pone.0071498)
Supplement: Table S1 — Biovariability Measures for Cellular Phenotypes. (DOCX) [file pone.0071498.s003.docx]

**Table S1.** Biovariability Measures for Cellular Phenotypes.

| **Measure** (*n*=20) | **Mean (SD)** | **% CVi** | **% CVg** | **II** | **CVg/**  **CV_total_** |
| --- | --- | --- | --- | --- | --- |
| %CD4^+^ Naive Cells | 38.3 (14.3) | 16.25 | 36.89 | 0.44 | 0.69 |
| %CD4^+^ Memory Cells | 46.5 (13.4) | 12.71 | 28.41 | 0.45 | 0.69 |

Data are means (SD). CVi, intra-person coefficient of variation; CVg, inter-person coefficient of variation; II, index of individuality (CVi/CVg); CV_total_=CVi+CVg.
